# Anti-CD20 treatment effectively attenuates cortical pathology in a rat model of widespread cortical demyelination

**DOI:** 10.1186/s12974-021-02189-w

**Published:** 2021-06-15

**Authors:** Michaela T. Haindl, Muammer Üçal, Benjamin Klaus, Lennart Tögl, Jana Dohrmann, Milena Z. Adzemovic, Christian Enzinger, Sonja Hochmeister

**Affiliations:** 1 Department of Neurology, Medical University of Graz, Graz, Austria; 2 Department of Neurosurgery, Research Unit Experimental Neurotraumatology, Medical University of Graz, Graz, Austria; 3 Department of Clinical Neuroscience, Center for Molecular Medicine, Karolinska Institutet, Stockholm, Sweden; 4 Center of Neurology, Academic Specialist Center, Stockholm Health Services, Stockholm, Sweden

**Keywords:** Progressive multiple sclerosis, Anti-CD20 therapy, Rat model

## Abstract

**Background:**

Cortical demyelination represents a prominent feature of the multiple sclerosis (MS) brain, especially in (late) progressive stages. We recently developed a new rat model that reassembles critical features of cortical pathology characteristic to progressive types of MS. In persons affected by MS, B-cell depleting anti-CD20 therapy proved successful in the relapsing remitting as well as the early progressive course of MS, with respect to reducing the relapse rate and number of newly formed lesions. However, if the development of cortical pathology can be prevented or at least slowed down is still not clear. The main goal of this study was thus to increase our understanding for the mode of action of B-cells and B-cell directed therapy on cortical lesions in our rat model.

**Methods:**

For this purpose, we set up two separate experiments, with two different induction modes of B-cell depletion. Brain tissues were analyzed thoroughly using histology.

**Results:**

We observed a marked reduction of cortical demyelination, microglial activation, astrocytic reaction, and apoptotic cell loss in anti-CD20 antibody treated groups. At the same time, we noted increased neuronal preservation compared to control groups, indicating a favorable impact of anti-CD20 therapy.

**Conclusion:**

These findings might pave the way for further research on the mode of action of B-cells and therefore help to improve therapeutic options for progressive MS.

**Supplementary Information:**

The online version contains supplementary material available at 10.1186/s12974-021-02189-w.

## Background

The involvement of B-cells in multiple sclerosis (MS) has received increasing attention in the past few years following the success of B-cell-targeted therapy. While the specific contribution of distinct subsets of B-cells to MS pathology remains unknown, in vitro experiments and animal studies pointed towards regulatory and inflammatory roles of several B-cell subsets, especially CD20 (cluster of differentiation 20) expressing cells [1, 2]. Treatment of MS patients in the relapsing-remitting disease phase (RRMS) with anti-CD20 therapy resulted in a significant reduction of newly formed brain lesions and clinical relapses. This indicated an additional antibody-independent and pro-inflammatory function of B-cells [2], by which they contribute to MS development and progression through targeting autoantigens, beside humoral antibodies, binding to brain cells, and thereby leading to tissue injury. Recent research also discussed leptomeningeal B-cell clusters to promote neuronal degeneration and demyelination, particularly in the later, progressive stages of the disease [3].

The underlying pathogenic mechanisms of RRMS and progressive MS (PMS) differ. RRMS is characterized by inflammation and demyelination primarily driven by adaptive immunity, while in PMS, innate immune cells such as macrophages, dendritic cells, microglia, and natural killer cells also play major roles, altogether emphasizing the multifaceted complexity in PMS pathogenesis. This difference could partially explain the fact that immunomodulatory or immunosuppressive drug formulations that successfully improve RRMS have been fairly ineffective in the treatment of PMS [4, 5]. Furthermore, chronic inflammation behind a closed blood-brain barrier (BBB) accompanied by microglial activation and continued involvement of T-cells and B-cells represent hallmark of PMS. However, clonally expanded plasma cells from MS patients produce antibodies directed against neurons and astrocytes but rarely against myelin components, suggesting that metabolic and energetic stress induced by inflammation could in fact precede demyelination and impede remyelination. Nevertheless, these antibodies caused demyelination in spinal cord explants in vitro, indicating an antibody-mediated pathology [6, 7].

Prior to clinical trials, the positive effect of B-cell depletion on lesion formation had been mostly studied in classical animal models of experimental autoimmune encephalomyelitis (EAE), a model epitomizing the human pathology of RRMS quite well [8–10]. Although a beneficial effect of anti-CD20 therapy has also been discussed for PMS, the lack of available animal models resembling the respective pathological features have so far hindered a better understanding of B-cell involvement in demyelinating lesions of progressive MS types.

We have recently established an animal model that reconstitutes cortical demyelination characteristic for the progressive MS brain, resulting in widespread subpial demyelination [11]. In the present work, we investigated the effects of anti-CD20 treatment on cortical MS pathology using two different approaches in our model. In the first approach, we treated the animals with anti-CD20 *after* immunization against a recombinant myelin oligodendrocyte glycoprotein (MOG1-125). This experimental setup served as reenactment of the situation in MS patients under anti-CD20 therapy with already existing intrathecal antibodies, which diminishes all CD20+ B cell populations except those at the undifferentiated and fully differentiated stages (plasma cells), leaving the already established antibody titers unchanged. In the second approach, the rats were treated with the anti-CD20 antibody *before* MOG immunization, to check whether CD20+ B-cell populations play a role in building the autoimmune response itself and whether intrathecal antibodies are involved in the demyelination process. Control groups of both approaches were treated with respective isotype matched control antibodies.

## Methods

### Animals

Adult (10–12 weeks of age) male Dark Agouti (DA) rats (*n* = 30), obtained from a commercial vendor (Janvier, France), were housed in the animal facility of the Biomedical Research Institute at the Medical University of Graz under standard conditions with 12 h light/dark cycle. Food and water were provided ad libitum. Animals’ health status was checked at least once daily by qualified personnel. Following catheter implantation, they were moved to modified high top single cages to avoid damage to the catheter. All animal experiments were carried out under approval of the local authorities (Bundesministerium für Wissenschaft und Forschung; 66.010/0195-V/3b/2018). Animal numbers are listed in Table 1 in detail.

**Table 1:** Number of animals per group

Experimental group		Number of DA rats *n* =
C0	Control, MOG immunized, no cytokine	4
C1	MOG before control antibody	4
E1	MOG before anti-CD20 therapy	10
C2	Control antibody before MOG	3
E2	Anti-CD20 therapy before MOG	8
In total		**29**

**Table 2:** Asymptotic significances calculated for the different groups via Mann-Whitney U test

Compared groups		PLP		Iba1		GFAP		Caspase-3		NeuN	
		Ipsi.	Contr.	Ipsi.	Contr.	Ipsi.	Contr.	Ipsi.	Contr.	Ipsi.	Contr.
**C0**	**E1**	0.198	0.230	0.317	0.391	0.647	0.200	1.000	0.721	0.156	0.063
**C0**	**E2**	0.172	0.181	0.174	0.220	0.163	0.092	0.401	0.746	0.606	0.366
**C0**	**C1**	**0.020**	**0.014**	**0.011**	**0.011**	**0.014**	**0.014**	**0.011**	**0.011**	**0.019**	**0.010**
**C0**	**C2**	**0.032**	**0.019**	**0.020**	**0.020**	0.053	0.053	**0.020**	**0.020**	**0.020**	**0.020**
**E1**	**E2**	0.195	0.106	0.416	0.817	0.517	0.746	0.524	0.772	0.074	0.083
**E1**	**C1**	0.392	0.088	**0.008**	**0.008**	**0.011**	**0.010**	**0.008**	**0.008**	**0.011**	**0.008**
**E1**	**C2**	0.300	**0.039**	**0.016**	**0.017**	**0.046**	**0.044**	**0.017**	**0.017**	**0.014**	**0.017**
**E2**	**C1**	**0.007**	**0.014**	**0.006**	**0.007**	**0.006**	**0.007**	**0.007**	**0.006**	0.062	**0.007**
**E2**	**C2**	**0.014**	**0.014**	**0.014**	**0.014**	**0.036**	**0.037**	**0.014**	**0.014**	**0.014**	**0.014**
**C1**	**C2**	1.000	0.724	0.724	0.372	1.000	0.064	1.000	0.724	0.157	0.724

*P*-values < 0.05 were considered to be significant. Significant *p*-values are given in bold

The planed animal groups were C1 = 5, E1 = 10, C2 = 5, and E2 = 10. We had one animal loss during surgery, resulting in a total animal number of *n* = 29. During the experiment, some catheter losses do occur. Those animals (*n* = 3) were switched to the C0 group, since the catheter losses occurred before any cytokine injection and we know from previous work that demyelination only appears when animals receive both MOG immunization and cytokine injection. The catheter losses are also the reason for the unequal partitioning.

### Experimental setup and groups

**Fig. 1:**
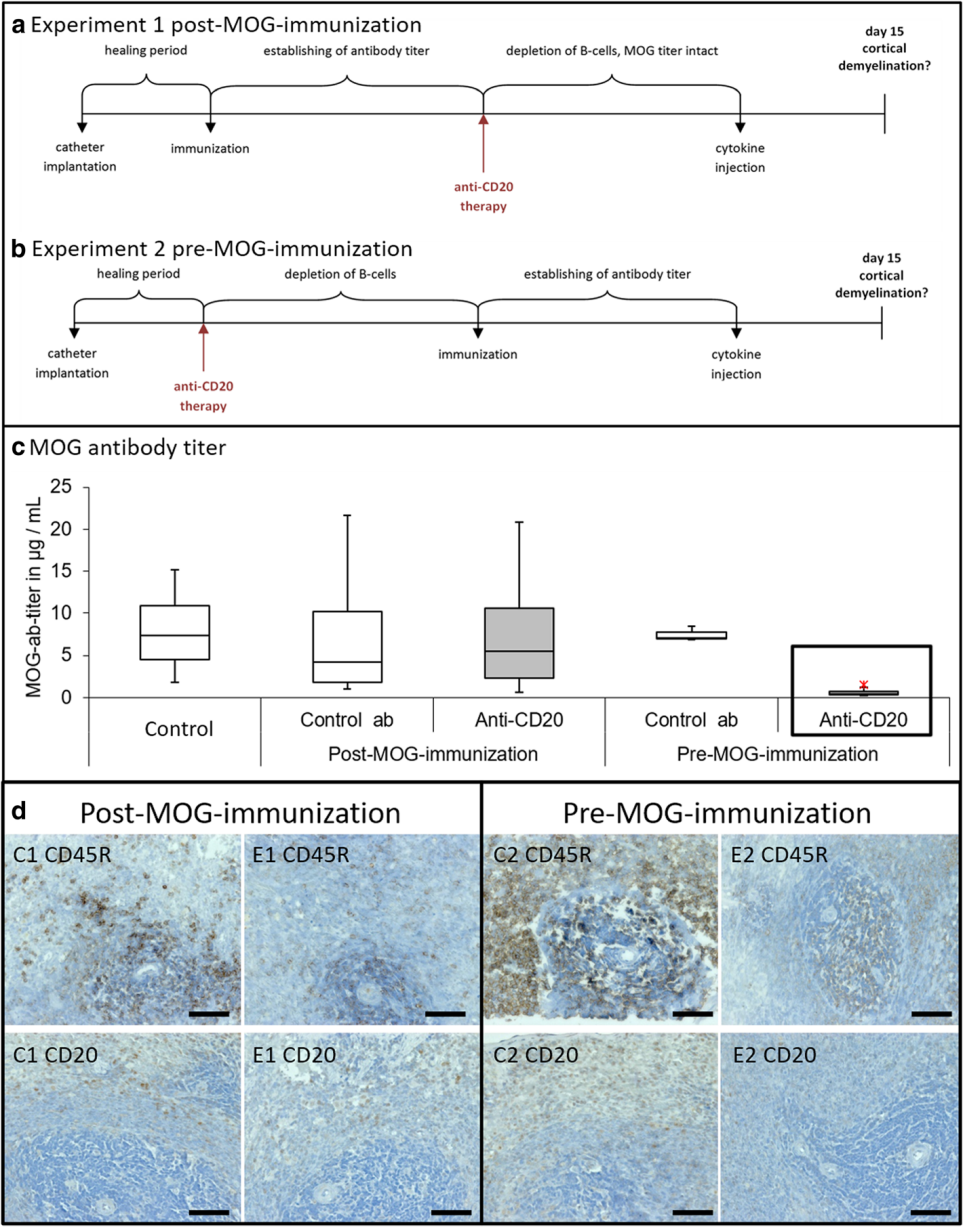
Timelines of the two different experimental approaches, MOG titer of the different experimental groups, and immunohistochemical staining of the spleen with two different B-cell markers. Experiment 1 is shown in **a**, where B-cell depletion is induced after catheter implantation and immunization. The MOG antibody titer is already established at that time. The hypothesis behind this approach is, that after cytokine injection through the catheter, cortical demyelination in our rats is at least reduced. In **b**, experiment 2 is schematically illustrated. Rats are treated with anti-CD20 therapy after catheter implantation but before immunization with MOG. The hypothesis is, that after treatment with anti-CD20 antibodies, less or no demyelination is expected. In both experiments, the healing period takes approximately 14 days of time and the establishing of MOG antibody titer approximately 4 weeks. The arrows indicated as “anti-CD20 therapy” mean one set of 2 injections with a time span of 7 days in between of either anti-CD20 antibodies or isotype control antibodies. In **c**, the MOG titer of the different groups is shown in μg/mL, with the results being in a comparable range (5–10 μg/mL) with no significant differences in between, except E2. As expected, only the E2 group, highlighted with a box around this group, shows significant differences to C0 (*p* = 0.017), C2 (*p* = 0.014), and E1 (*p* = 0.004). The asterisk indicates an outlier. The immunohistochemical stainings of spleen (**d**) show pictures near lymph follicle, opposed are control groups (C1 and C2) and experimental groups (E1 and E2), respectively. The first line shows CD45R positive cells and the second line represents CD20 positive cells (positive cells appear in brown). Both markers show more positive cells in C1 and C2 in comparison to E1 and E2, as expected. In additional Figure 2 (e, f), a quantification of CD20 and CD45R positive cells in lymph follicles is represented with significant differences between experimental groups and control groups (*p* < 0.009). Scale bars represent 50 μm

All animals underwent intracerebral catheter implantation, as described previously [11]. After 2 weeks of healing of the blood-brain barrier (BBB), animals were immunized with a recombinant MOG1-125 protein and received antibody treatment (anti-CD20 or isotype control) in two alternative orders: In experiment 1, animals received anti-CD20 (E1, *n* = 9) or isotype control antibody (C1, *n* = 4) *after* MOG immunization. In experiment 2, rats were treated with anti-CD20 (E2, *n* = 8) or isotype control antibody (C2, *n* = 3) *before* MOG immunization. Control animals (C2) received the isotype control antibody (*n* = 3). Another group of control animals were implanted and immunized, but did not receive antibody nor cytokine (C0, *n* = 4). The timelines in Fig. 1(a) and (b) gives an overview of the two approaches along with the crucial experimental steps.

### Catheter implantation

Animals were anaesthetized with intraperitoneal administration of a mixture of 0.03 mg/kg Fentanyl (Jansen-Cilag Pharma, Vienna, Austria), 0.6 mg/kg Midazolam (Erwo Pharma, Brunn am Gebirge, Austria), and 0.3 mg/kg Medetomidin (Orion Pharma, Espoo, Finland). Animals were well fixed on a stereotactic frame (David Kopf Instruments, Tujunga, CA, USA), where temperature stability was controlled using a rectal thermometer and a heating pad. A longitudinal incision was applied to head skin and the skull was exposed by removal of periosteum. Bregma point was identified by visual inspection after removing the skin and tissue by scraping with a scalpel and disinfecting the area with an alcoholic solution. A cranial hole with a diameter of 0.5 mm was drilled for the catheter over the right parietal cortex, 2.0 mm from the bregma and 2.4 mm from the medial suture. Three cranial holes with a diameter of 1 mm were drilled for screws, over the right and left parietal cortex at a few millimeters of distance from the first hole and plastic screws (PlasticsOne, Roanoke, VA, USA) were tightened by 2–3 full turns. The catheter (PlasticsOne, Roanoke, VA, USA) was cut to 2.0 mm length, inserted into the first opening and was placed into the cerebral cortex with the tip just above the corpus callosum. Catheter and screws were fixed with the help of dental cement (Heraeus Kulzer, Hanau, Germany). Afterwards, the skin was closed with resorbable sutures and anesthesia was antagonized with subcutaneous administration of a mixture of 0.105 mg/kg Flumazenil (Anexate; Roche Austria, Vienna, Austria) and 0.63 mg/kg Atipamezol (Antisedan; Orion Pharma). Animals were subcutaneously injected with enrofloxacin on the day of surgery (dose 7.5 mg/kg body weight; Baytril; Provet AG, Lyssach, Switzerland). The day after surgery, animals received another dose of enrofloxacin as well as a dose of (2% (v/v)) Carprofen s.c. (Rimadyl; Zoetis Austria, Vienna, Austria) in physiological saline under brief anesthesia with isoflurane (AbbVie, Vienna, Austria) to control pain. Post-surgical recovery was uneventful in all cases. After 14 days, the catheter was completely healed in and the BBB was restored again. The integrity of the BBB was verified in previous experiments during establishing and validation of the animal model by lack of fibrin leakage in the tissue after 14 days. This was assessed by immunohistochemistry of the catheter area after different time points after catheter implantation [11].

### MOG immunization

Myelin oligodendrocyte glycoprotein (MOG, amino acids 1–125 from the N-terminus) used for EAE induction was expressed in *Escherichia coli* and purified to homogeneity by chelate chromatography [12]. The purified protein, dissolved in 6 M urea, was dialyzed against phosphate-buffered saline (PBS) to obtain a physiological preparation that was stored at − 70 °C. Rats were briefly anaesthetized by inhalation of isoflurane and injected subcutaneously at the base of the tail with a total volume of 200 μl of 5 μg MOG diluted in saline, emulsified in incomplete Freund’s adjuvant (IFA; Sigma-Aldrich, Buchs, Switzerland). For the assessment of anti-MOG titers, peripheral blood (approximately 400 μL) was collected from the tail tip four weeks after immunization. Serum was harvested by 10 min centrifugation at 4000×*g* using a desktop centrifuge and stored at – 80 °C until usage.

### Anti-CD20 treatment

Animals were briefly anaesthetized by inhalation of isoflurane and injected intravenously at the base of the tail with 200 μL of 5 mg/ml rat-specific research antibody preparation (aCD20AB, Genentech, Roche Group, anti-mCD20 mlgG2a 5D2) twice, with a time span of 7 days in between. Control animals were injected with the isotype control (CAB; anti-mouse IgG_2a_ƙ, Leinco Technologies, MO, USA) at the same volume and concentration.

We set up two different approaches in order to attain our research goals (as shown in Fig. 1a and b). Both approaches were divided into two further groups where we used the control antibody (CAB) instead of the aCD20AB.

### Assessment of MOG titer

MOG titers were assessed using enzyme linked immunosorbent assay (ELISA). In short, MOG (5 μL MOG/ml PBS) was coated on a 96-well plate (Nunc, Wiesbaden, Germany) and incubated for 1 h at 37 °C. Then, the plate was blocked with 1% bovine serum albumin (BSA; Serva, Heidelberg, Germany) in PBS for 1 h at room temperature. Afterwards, the plate was incubated with rat sera (1:50) and standard for 2 h at 37 °C. IgG specific horseradish peroxidase conjugated anti-rat antibody (1:10,000) was used for detection. 2,2′-Azino-bis 3-ethylbenzthiazoline-6-sulfonic acid (ABTS) was added as substrate. Between these steps, the plate was washed three times with PBS/Tween. The optical density was measured at 405 nm wavelength.

### Opening of the BBB

The BBB was opened by the time animals developed a MOG titer of 5-10 μg/mL or more, as assessed via ELISA. In the original publication of this animal model [11] with control animals for each experimental step, we show that cortical demyelination only occurs, when both — MOG immunization and cytokine injection — is performed. Omitting either of these steps, there is no demyelination observable, so our model has an intrinsic correction whether BBB opening worked or not, so in the current study there was no additional methodology for verification used. All animals received 2 μL of a cytokine mixture containing 500 μg/mL TNF-alpha (R&D systems, Abington, UK) and 300 U recombinant rat IFN-gamma/μL (PeproTech, London, UK), using a specific programmable syringe pump through the catheter (injection rate of 0.2 μL/min).

### Sacrificing and tissue harvesting

Tissue samples were collected on day 15 after cytokine injection, at a time when we previously have shown that the cortical pathology reaches its maximum [11], after euthanasia using overdose anesthesia by intraperitoneal injection of 1 ml pentobarbital sodium (Richter Pharma, Austria). Under deep anesthesia, the thorax was opened and the animals were transcardially perfused with 4% formaldehyde (Merck, Darmstadt, Germany) in phosphate-buffered saline (PBS, pH 7.4). Brains, spinal cords, and spleens were dissected and post-fixed in 4% FA for 24 hours at 4 °C.

### Neuropathology, immunohistochemistry, and immunofluorescence

After routine embedding in paraffin, brains, spinal cords, and spleens were cut in 1.5 μm sections. For immunohistochemistry (IHC), sections were dewaxed in xylol (Fisher Thermo Scientific, Schwerte, Germany), rehydrated, and steamed for 1 h in citric acid (Merck) for antigen retrieval using a commercial steamer [11, 13]. To avoid unspecific binding, sections were blocked with 2.5% normal horse serum (Vector Laboratories Burlingame, CA, USA) at room temperature for 20 min, prior to incubation with the primary antibodies at 4 °C overnight. For a detailed list of the used antibodies and dilutions, see the respective additional Table 1 in the appendix. The enzymatic ImmPRESS System was utilized for detection with 3,3′diaminobenzidine-tetrahydrochloride (DAB, Sigma-Aldrich, Buchs, Switzerland) and counterstaining was performed with hematoxylin. After dehydration, slices were covered with a xylene based mounting medium (Shandon Consul-Mount, Fisher Thermo Scientific) and a coverslip. The slices immunostained with fluorescently labeled antibodies were washed with PBS after the incubation with fluorescent labeled secondary antibody (VectaFluor, Vector Laboratories) and covered with an aqueous mounting medium (Vectashield, Vector Laboratories) and a coverslip.

### Quantitative histopathological evaluation

For the quantification of demyelination, microglial activation, astrocytic reaction, apoptotic cells, and neurons, serial sections of the catheter insertion area were used. Demyelination was assessed by quantification of cortical loss of proteolipid protein (PLP) immunoreactivity for each hemisphere, using an optical grid at a magnification of × 200; values were then transformed to mm^2^. Activated microglia (Iba-1), activated astrocytes (GFAP), apoptotic cells (Caspase-3), and neurons (NeuN) were assessed in three full optical grids in the cortex per hemisphere at a magnification of × 200 and average values were then converted to cells/mm^2^. Quantifications were performed by one blinded investigator.

### Statistical analyses

All statistic calculations were performed using SPSS Statistics software (v23, IBM, USA); graphs were generated using Microsoft Excel 2010. All data is presented in box-whisker plots. We tested for normal distribution of the data by performing the Kolmogorov-Smirnov test with most of the variables being not normally distributed. Statistical significance of the observed differences was assessed with the Kruskal-Wallis H-Test, followed by a Mann-Whitney U test for pairwise comparisons. A difference with a *p*-value ≤ 0.05 was deemed statistically significant.

## Results

### Anti-CD20 treatment had an impact on anti-MOG titers

As expected, anti-CD20 treatment had a significant influence on building of the anti-MOG titers (χ^2^(4) = 11.6, *p* < 0.02). A pairwise comparison showed that anti-CD20 treatment before the MOG immunization resulted in significant attenuation in anti-MOG titers in comparison to the C0 control animals (*p* = 0.017), isotype control group (C2) (*p* = 0.014) as well as to the group that received anti-CD20 treatment after the MOG immunization (E1) (*p* = 0.004), while a comparable range of anti-MOG titers were observed in all other groups (Fig. 1c). Nevertheless, post-mortem analyses of CD45R- and CD20-immunoreactivities were equally diminished in both anti-CD20 treated groups in comparison with the controls that were treated with isotype control antibody, regardless of the timing of treatment (Fig. 1d).

#### Anti-CD20 treatment attenuates cortical demyelination

**Fig. 2:**
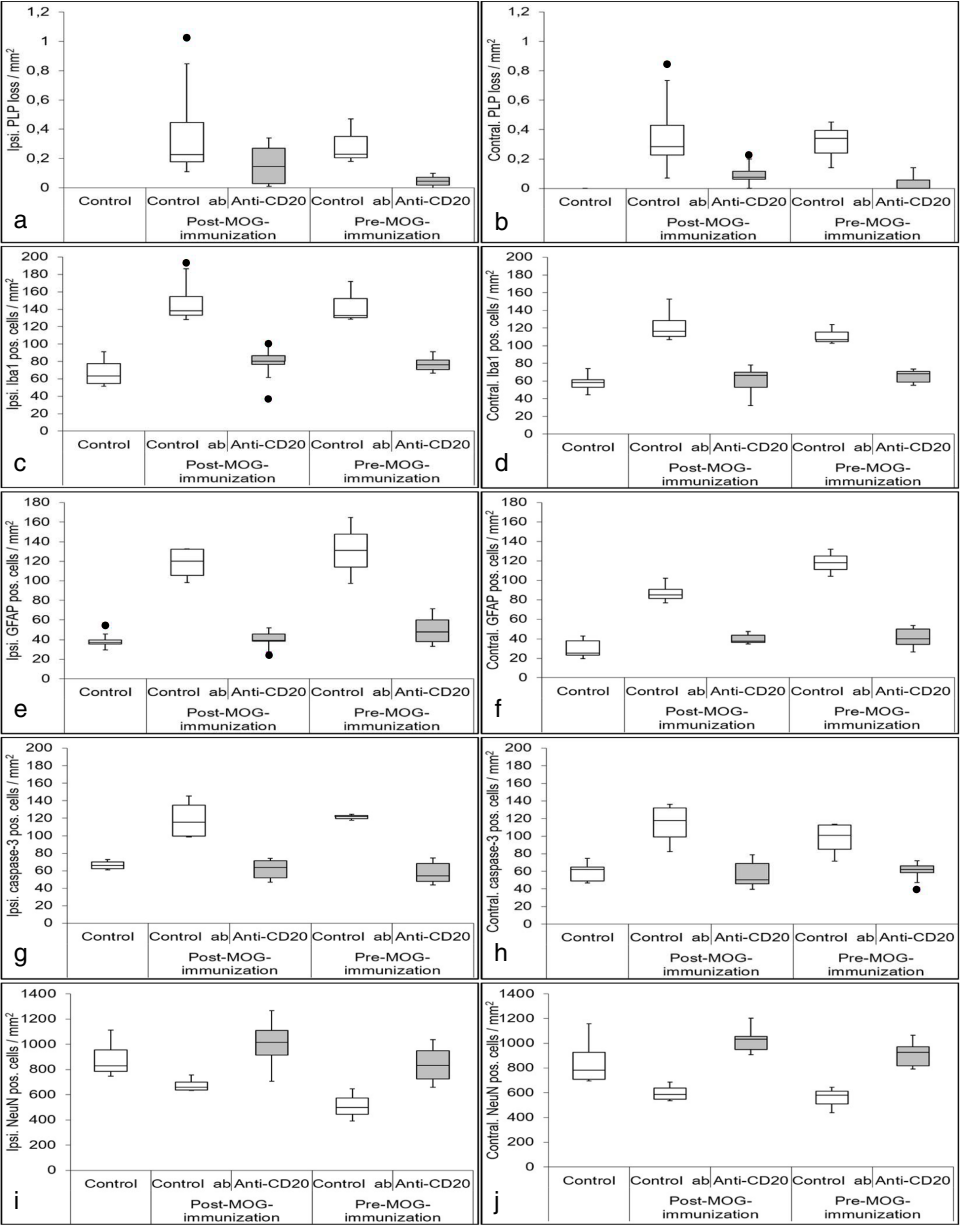
Quantification of cortical demyelination, microglial activation, astrocyte activation, apoptotic cells, and neuronal loss. PLP loss is illustrated in **a** on the ipsilateral hemisphere and in **b** on the contralateral hemisphere. Both anti-CD20 therapy approaches result in less PLP loss, with a slightly higher impact of E2. Microglial activation is shown in **c** on the ipsilateral hemisphere and in **d** on the contralateral hemisphere. Both anti-CD20 therapy approaches show less microglial activation compared to the control groups. The microglial activity is comparable to the C0. Results of activated astrocytes are shown in **e** on the ipsilateral and in **f** on the contralateral side. There are more activated astrocytes detectable in control groups in comparison to the anti-CD20 therapy groups, which are comparable to the C0. Apoptotic cell counts are illustrated in **g** on the ipsilateral and in **h** on the contralateral side. On both sides, apoptotic cell counts are reduced compared to the control groups and showed comparable cell counts as determined for the C0s. Quantification of neurons is shown in **i** on the ipsilateral and in **j** on the contralateral hemisphere. Control groups showed higher neuronal loss in comparison to C0s and anti-CD20 therapy groups. In all of those quantifications, there is a significant difference between the C0 and the control groups, but there is no significant difference between C0 and anti-CD20 therapy groups, indicating an overall favorable role of this therapy approach. Black dots indicate outlier. The data of all significances between all tested groups is given in Table 2

**Fig. 3:**
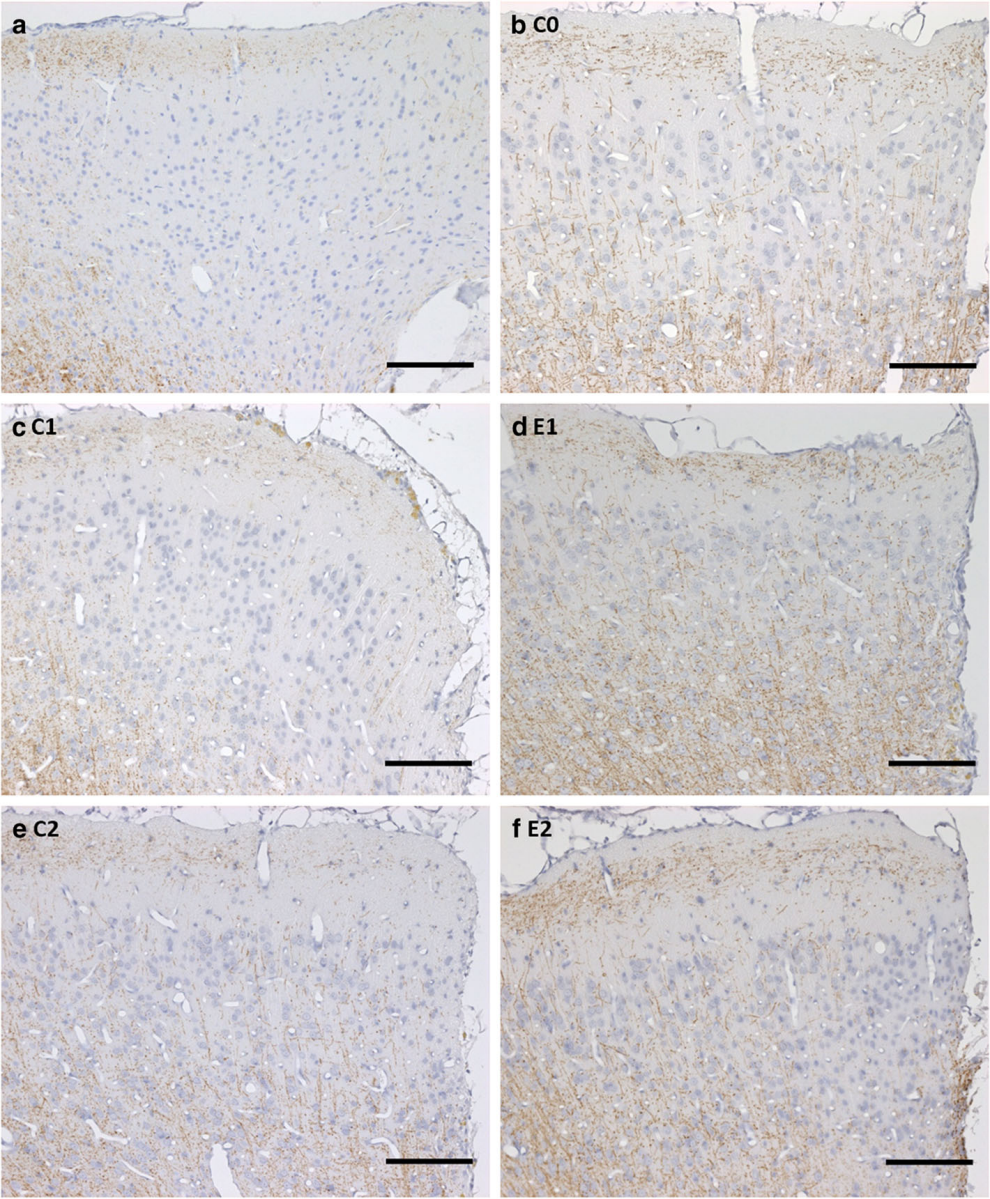
Representative immunohistochemical PLP staining comparing the two experimental approaches to C0 and an animal receiving no therapy at all. One upper corner of the catheter puncture side on the ipsilateral hemisphere is shown on each photo. Positive staining appears in brown, counterstained cell nuclei in blue. The first line shows the difference between a rat receiving no therapy at all (**a**) with the most cortical demyelination detectable and a C0 (**b**) with no PLP loss at all. In the second line, the C1 group (**c**) shows more PLP loss than the therapy group E1 (**d**). This pattern is also comparable in the last line, showing C2 (**e**) and E2 (**f**). E1 and E2 show results comparable to C0. Scale bars represent 100 μm

Animals treated with an isotype control antibody before or after MOG immunization showed a statistically significant cortical demyelination, substantiated by the reduction of PLP-immunoreactivity in both cerebral hemispheres, at 15 days after cytokine injection in comparison with the controls without cytokine injection (C1 ipsilateral (ipsi): *p* < 0.02, C1 contralateral (contra): *p* < 0.01, C2 ipsi: *p* < 0.03, C2 contra: *p* < 0.02) (Fig. 2a, b). Anti-CD20 treatment, however, prevented the cytokine-induced myelin loss to a large extent in both experimental groups (E1 ipsi: *p* < 0.20, E1 contra: *p* < 0.23, E2 ipsi: *p* < 0.17, E2 contra: *p* < 0.18; with no significant difference detectable between controls without cytokine injection and treatment group). It is worth to note that the preserved PLP-immunoreactivity was slightly stronger in the group that was treated with anti-CD20 before the MOG immunization (E2), when compared to those treated afterwards (E1) but not reaching statistical significance (ipsi: *p* < 0.195, contra: *p* < 0.106). Representative images of PLP immunostaining are shown in Fig. 3. Since the cortical demyelination is a major outcome measure, we provide another quantification of other myelin proteins, namely MBP and MOG in additional Figure 2. Both markers show a significant difference between E2 to C1 and C2 on both sides (MBP ipsi. C1: *p* < 0.01; C2: *p* = 0.04; MBP contra. C1: *p* < 0.01; C2: *p* = 0.03; MOG ipsi. C1: *p* < 0.01; C2: *p* = 0.02; MOG contra. C1: *p* < 0.01; C2: *p* = 0.02). The MOG quantification also showed significant differences between E1 to C1 and C2 on both sides (MOG ipsi. C1: *p* < 0.02; C2: *p* = 0.02; MOG contra. C1: *p* < 0.01; C2: *p* = 0.02). All significances for MBP and MOG quantification are listed in additional Table 2. As reported in our previous publication [11], there are hardly any T- and B-cell infiltrates in this animal model; only traces of cellular infiltrates can be found in the meninges. Representative photos are given in additional Figure 1.

#### Anti-CD20 treatment alleviates inflammatory reaction in cortex

**Fig. 4:**
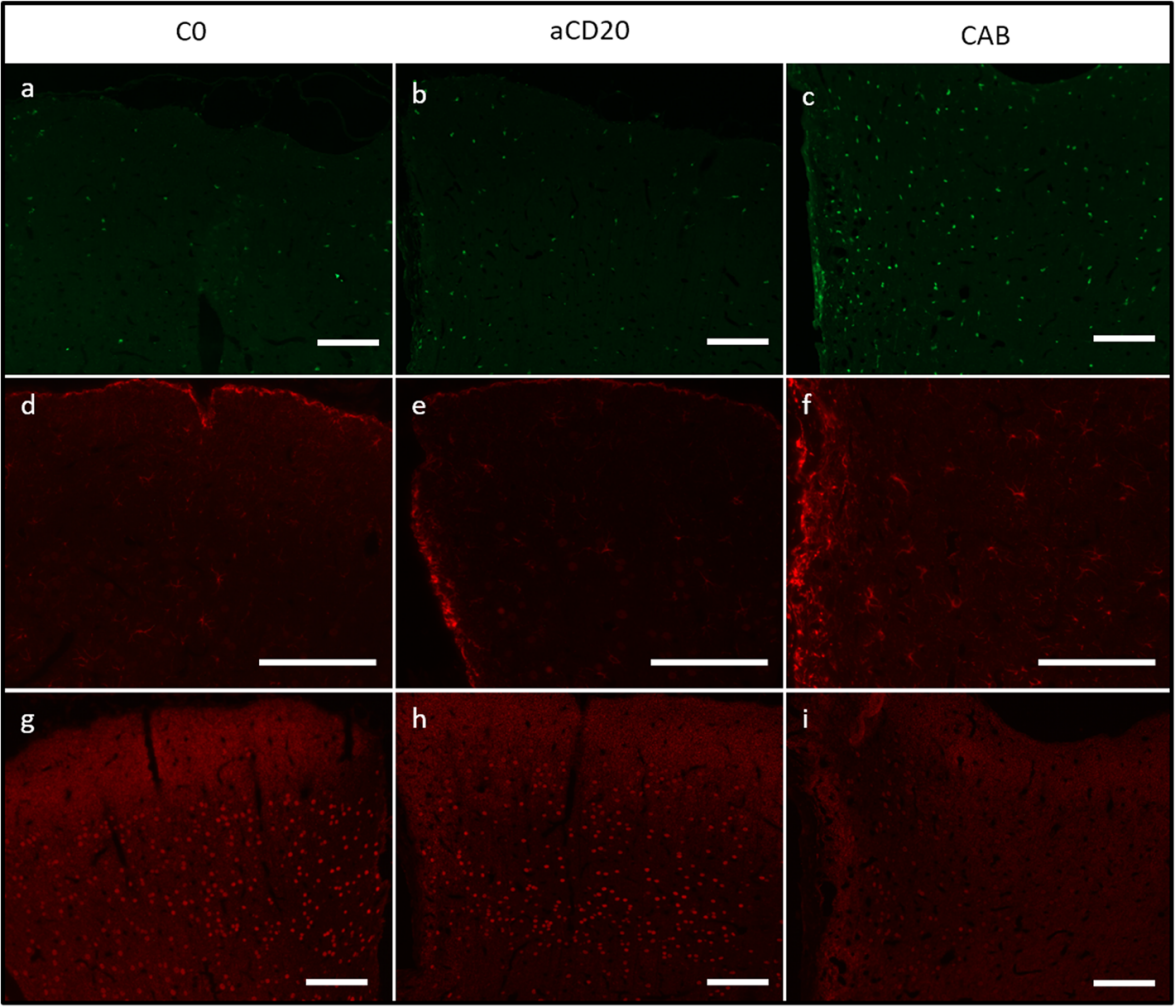
Representative immunofluorescent stainings comparing the therapy groups, control groups, and C0. Because of the comparability of the different experimental approaches, only C2/E2 results are shown side by side for comparison to avoid redundancy and allow an easy overview. The first line shows Iba1 staining (marked in green), a marker for microglial activation. Microglial activation of C0 (**a**) is comparable to E2 (**b**), whereas C2 (**c**) shows more microglial activation. A similar pattern is detectable when looking at GFAP staining (marked in red), a marker for activated astrocytes in the second line. Also, C0 (**d**) and E2 (**e**) results are comparable and there are more activated astrocytes detectable in C2 (**f**). The neuronal preservation (red dots) in E2 (**h**) is comparable to neuron occurrence in naïve animals (**g**) in C2; however, neuronal loss is detectable (**i**). Scale bars represent 100 μm

Intracerebral cytokine injection into the animals that were treated with isotype control antibodies resulted in significant increases in the number of activated (Iba1+) microglia in both hemispheres compared with the C0 controls (C1: *p* < 0.01; C2: *p* = 0.02) (Fig. 2c, d). Concordant increases were observed in the number of reactive astrocytes (C1: *p* < 0.02; C2: *p* = 0.05) (Fig. 2e, f). Anti-CD20 treatment, however, effectively attenuated microglial activation (E1: *p* < 0.01, E2: *p* = 0.02) and astrocytic reaction (E1: *p* < 0.01, E2: *p* = 0.04) in both ipsi- and contralateral cerebral cortices in comparison with the corresponding controls. Together, these findings suggest that anti-CD20 treatment prevented cytokine-induced cortical demyelination through suppression of inflammatory responses. Representative immunofluorescent images of microglial activation are shown in Fig. 4a-c and of astrocytic reaction in Fig. 4d-f, respectively.

#### Anti-CD20 treatment reduced apoptotic cell death and neuronal loss

We have previously shown that the apoptotic cell death and neuronal loss were two other consequences of intracerebral cytokine injection following MOG immunization in our model [11]. In the current study, we observed significant increases in the number of Caspase-3+ cells in the cerebral cortices of animals that had been treated with isotype control antibody compared to the animals without a cytokine injection (both ipsi- and contralaterally; C1: *p* < 0.01, C2: *p* = 0.02) (Fig. 2g, h). Representative histological pictures of Caspase-3 immunohistochemistry are represented in additional Figure 1 c-f. Most of the apoptotic cells are astrocytes, as described previously in Ücal et al. [11]. In additional Figure 1 g and h, representative immunohistological double stainings of GFAP+/Caspase-3+ cells are shown. Furthermore, there was a concordant reduction in cortical NeuN immunoreactivity in these animals (ipsilateral: *p* < 0.02 for C1 and C2; contralateral: *p* < 0.01 for C1 and *p* = 0.02 for C2). Anti-CD20 treatment, on the other hand, prevented the apoptotic cell death following a cytokine injection in the cerebral cortices in both treatment groups compared with the corresponding controls (both ipsi- and contralaterally; E1: *p* = 0.008, E2: *p* = 0.01). NeuN immunoreactivity, moreover, was preserved in both experimental groups (Fig. 2i, j). In none of the groups, a significant difference could be detected between C1 and C2 (*p*-values range between 0.06 and 1.00, see additional Table 2 for details). Representative pictures of neuronal distribution via immunofluorescent staining are shown in Fig. 4g-i.

## Discussion

Recent research has shown that B-cell depletion suppresses lesion development in experimental antibody-independent models of MS; therefore, B-cells seem to play an important role in white matter lesion development, independent of auto-antibody production. There is also evidence for B-cells acting as antigen-presenting cells (APCs) and their involvement in the development and progression of MS appears likely. Anti-CD20 therapy can reduce the proliferation and activation of autoreactive CD4+ T-cells and IL-17 production in the CNS, resulting in a reduced clinical severity in animal models of MS [9, 14, 15]. In our model of progressive MS, we demonstrated that administration of anti-CD20 therapy before or after immunization against MOG attenuated cortical demyelination induced by intracerebral injection of inflammatory cytokines. However, it should be noted that the effect was more pronounced in the group treated with anti-CD20 *before* the MOG immunization (E2 group). The anti-MOG-antibodies do play a role in our model and other experimental animal models like EAE [16], and depletion of B-cells effectively suppresses the building of immunization against MOG.

MOG immunization in DA rats per se frequently resulted in a slight temporary inflammation of limb joints, which spontaneously resolved in a couple of weeks. None of the anti-CD20 treated animals, however, showed such a reaction throughout the course of the experiments, indicating an anti-CD20-mediated suppression of overall peripheral autoimmune reactions, which are triggered by immunization similar to other autoimmune disease models [17–19]. It should, however, be noted that in all experimental groups, intracerebral injection of cytokines took place once the MOG titers reached a threshold level (6 μg/ml in most animals of the group). Therefore, the observed effects did not solely depend on suppression of antibody production, although the prognostic clinical severity closely correlates to antibody titers in various autoimmune disease models, like EAE [16]. Our findings allow us to conclude that the larger part of the mechanism is mediated via other B-cell functions, for example antigen presentation and induction of innate immune responses. Remarkable reductions observed in microglial activation and astrocytic reaction in anti-CD20 treated animals further substantiated these assumptions.

Even though the demyelination of isotype matched control antibody (C1 and C2) groups was invariably distinctive compared to the anti-CD20 treated groups, we found less demyelination in comparison to previous investigations in Ücal et al. [11]. The PLP loss in these controls was only between 0.4 ± 0.2 (C1) and 0.3 ± 0.1 (C2) per mm^2^ in comparison to animals in our previous experiments without any treatment where we observed a PLP loss of 1.0 ± 0.2 per mm^2^ on day 15. This effect is, presumably, attributable to a similar phenomenon already known from the studies that have reported a beneficial impact of unspecific immunoglobulin therapy (e.g., [20]). As a clinical standard therapy, unspecific antibodies obtained from blood preservations are used to treat several immune-mediated diseases to enable formation of unspecific complexes, which thereby inactivate pathogenic antibodies [21]. Our isotype control antibody might have acted in a similar way.

A common theory regarding cortical pathology in MS is the consideration of unnoticed cortical damage, starting early in the disease course besides the obvious white matter lesion formation, slowly accumulating over decades by successively exhausting the regenerative capacity. In patients entering a progressive MS phase, among other numerous tissue changes, damage to the cortex including cortical demyelination has accumulated. Our data support the assumption, that anti-CD20 therapy — administered early in the disease course — could reduce, delay or even prevent cortical demyelination, as well as reduce the extent of apoptotic cell death, thereby promoting neuronal survival. The remarkably similar results of our both experimental approaches involving anti-CD20 therapy indicate that, in this context, cellular functions of B-cells seem to be much more important to disease progression than antibody production alone. This means that anti-CD20 therapy seems to have a positive effect on disease course, even when administered after disease onset and not only before [16]. Given the wide use of CD20 antibody therapy in RRMS patients as well as primary progressive MS [22], time will tell if indeed the conversion rate to progressive MS or evolution of disability will diminish, as extrapolated from these observations. Additionally, this work proves the suitability of our animal model for mechanistic research.

For further research, it would be interesting to investigate if there is a benefit from anti-CD20 therapy during or even after initiation of multiple waves of demyelination in this animal model, which we have shown previously to result in a marked global brain atrophy [11]. Also, a comparison of our results to the anti-CD20 effect in other models of cortical demyelination [23–25] would further complete the investigation. Other models like the cuprizone model have also achieved cortical demyelination [25]. While it is way easier to induce cortical demyelination in rodents by feeding cuprizone compared to our long experimental setup involving surgery, our model has several advantages; firstly, we induce an inflammatory cortical demyelination instead of a toxic demyelination, and the implanted catheter allows access to the cortical brain tissue for both induction of multiple waves of demyelination by repeated injections of inflammatory cytokines as well as possibly the administration of putative therapeutic agents. However, as with all animal models in medical research, each one has a different focus and often only the use of different models can shed light on different aspects of a research question.

Investigation of molecular mechanisms behind our findings is a further research goal. A protein mapping especially of different cytokines in our model and overview of different genes related to TNFα and IFNγ signaling in the experimental groups compared to controls would further increase our understanding of how anti-CD20 treatment affects cortical pathology.

A limitation of our current study is the lack of longitudinal assessments such as MRI scans. In fact, we chose a magnetic resonance imaging (MRI) compatible catheter for all our experiments to allow us this option. In our original work [11], we present our MRI results (28 in vivo measurements and 7 ex vivo measurements; T2 weighted images), but unfortunately, we were not able to detect the cortical demyelination in MRI which we see in histology. In Ücal et al. [11], we discuss this problem as likely because of insufficient resolution of MRI to detect the very small rim of demyelination of the cortex of the rats. Also, even in human multiple sclerosis patients, whose brains are of course much larger than rats, cortical demyelination is very hard to detect in conventional MRI, and therefore often largely underestimated, although it contributes to overall disease progression and disability. In the present study, anti-CD20 treatment drastically reduced the extent of cortical demyelination, so the chances of detection with longitudinal MRI scans is even lower than in our previous study; therefore, we did not perform MRI in this study.

## Conclusions

Our results indicate a critical involvement of CD20+ B-cells on cortical lesion development, given the fact that in both experimental approaches cortical pathology was significantly attenuated upon anti-CD20 treatment, reflected in reduced myelin loss, astrocyte and microglial activation, apoptosis, and neuronal loss. Furthermore, we show that the B-cell populations had a significant role in building autoimmunity against MOG. Finally, our data demonstrate that already established intrathecal antibodies do not play a significant role in cortical demyelination in our animal model.

## Supplementary Information

**Additional file 1: Additional Table 1.** List of antibodies used in this study. **Additional Table 2.** Asymptotic significances calculated for MBP and MOG. uantification via Mann-Whitney-U test. *P*-values < 0.05 were considered to be significant (given in bold). **Additional Table 3.** Results and asymptotic significances calculated for CD20 and CD45R quantification in spleen via Mann-Whitney-U test. *P*-values < 0.05 were considered to be significant.

**Additional file 2: Additional Figure 1.** Supplementary immunohistochemical stainings. Part of a lymph follicle of C0 showing in **(a)** the B-cell marker CD45R and in **(b)** CD20. Representative immunohistochemical staining of Caspase-3 is shown in **(c) – (f)**. Positive, apoptotic cells appear in brown, there is no counterstaining visible. In comparison to the controls C1 **(c)** and C2 **(e)** there are hardly any apoptotic cells detectable in the therapy groups E1 **(d)** and E2 **(f)**. Immunohistochemical double staining of GFAP (violet) and Caspase-3 (brown) is shown in **(g)** and **(h).** Most of the apoptotic cells are astrocytes. There are much more apoptotic astrocytes detectable in controls **(g)** in comparison to the therapy group **(h).** Infiltrates with T- and B-cells are very sparse in this animal model. CD3 positive T-cells and CD19 positive B-cells were only detected in minor traces in the meninges. Representative pictures of T-cells in E1 are given in **(i)** and of B-cells in **(j)**. Red arrows point at the very few positive stained cells in dark brown. Scale bars represent 100 μm.

**Additional file 3: Additional Figure 2.** Quantification of additional myelin markers MBP and MOG and quantification of CD20 and CD45R positive cells in spleen. All myelin quantifications of additional markers show comparable results to PLP quantification. For MBP there are significant differences between E2 and C1 (ipsi.: *p* < 0.008; con.: *p* < 0.010) and E2 and C2 (ipsi.: *p* < 0.037; con.: *p* < 0.034) on both sides **(a)** and **(b)**. There are significant differences between all experimental and control groups on both sides for MOG **(c)** and **(d)** with *p* values ranging from 0.011 to 0.024. For exact *p*-values for all groups see additional Table 2. For both B-cell markers, CD20 **(e)** and CD45R **(f)**, there is a significant difference detectable between all experimental groups and control groups (*p* < 0.009, see also additional Table 3). There is no significant difference between E1 and E2 or C1 and C2. For these results a representative set of n=5 lymph follicle per group was quantified.

## Data Availability

Supporting data and information about used material can be accessed by contacting one of the authors.

## Abbreviations

APC: Antigen-presenting cells
BBB: Blood-brain barrier
DA: Dark Agouti
EAE: Experimental autoimmune encephalomyelitis
GFAP: Glial fibrillary acid protein
IFA: Incomplete Freund’s adjuvant
IHC: Immunohistochemistry
MBP: Myelin Basic Protein
MOG: Myelin oligodendrocyte glycoprotein
MS: Multiple sclerosis
MRI: Magnetic resonance imaging
PBS: Phosphate-buffered saline
PLP: Proteolipid protein
