# Supplementary material for: Anti-CD20 treatment effectively attenuates cortical pathology in a rat model of widespread cortical demyelination
Source: J Neuroinflammation. 2021 Jun 15;18:138. doi: 10.1186/s12974-021-02189-w (PMC8207776; doi:10.1186/s12974-021-02189-w)
Supplement: Supplementary file 1 — Additional file 1: Additional Table 1. List of antibodies used in this study. Additional Table 2. Asymptotic significances calculated for MBP and MOG. uantification via Mann-Whitney-U test. P-values < 0.05 were considered to be significant (given in bold). Additional Table 3. Results and asymptotic significances calculated for CD20 and CD45R quantification in spleen via Mann-Whitney-U test. P-values < 0.05 were considered to be significant. [file 12974_2021_2189_MOESM1_ESM.docx]

**Additional material**

**Additional table 1: List of antibodies used in this study**

| **Antibody** | **Target** | **Host** | **Dilution** | **Company** | **Catalog number** | **AB ID** |
| --- | --- | --- | --- | --- | --- | --- |
| Isotype control antibody | IgG2ak low endotoxin functional formulation, platinum | mouse | 2 mg/mL | Leinco Technologies | P381 | AB_2831654 |
| Caspase3 | Anti-active Caspase3 antibody | rabbit | 1:500 | Abcam | ab2302 | AB_302962 |
| GFAP | anti-GFAP Ab-6 (Clone ASTRO6) | mouse | 1:100 | Thermo Scientific | MS-1376 | AB_1095984 |
| Iba1 | Anti Iba1 | rabbit | 1:1000 | Wako | 019-19741 | AB_839504 |
| NeuN | Anti-NeuN, clone A60 (KC) | mouse | 1:100 | Millipore | MAB377-KC | AB_2298772 |
| PLP | Anti-myelin proteolipid protein | mouse | 1:500 | AbD Serotec | MCA839G | AB_2237198 |
| anti-rabbit ImmPRESS reagent | anti-rabbit IgG | horse | ready to use | Vector Laboratories | MP-7401 | AB_2336529 |
| anti-mouse ImmPRESS reagent | anti-mouse IgG | horse | ready to use | Vector Laboratories | MP-7422 | AB_2336527 |
| VectaFluor, DyLight 594 | anti-mouse IgG | horse | ready to use | Vector Laboratories | DI-2794 | AB_2336783 |
| VectaFluor, DyLight 488 | anti-rabbit IgG | horse | ready to use | Vector Laboratories | DI-1788 | AB_2336781 |

**Additional table 2: Asymptotic significances calculated for MBP and MOG quantification via Mann-Whitney-U test.** P-values < 0.05 were considered to be significant (given in bold).

| **Compared groups** | | **MBP** | | **MOG** | |
| --- | --- | --- | --- | --- | --- |
|  |  | **Ipsi.** | **Contr.** | **Ipsi.** | **Contr.** |
| **C0** | **E1** | **0.016** | **0.013** | **0.009** | **0.016** |
| **C0** | **E2** | **0.003** | 0.058 | **0.003** | **0.030** |
| **C0** | **C1** | **0.007** | **0.014** | **0.007** | **0.014** |
| **C0** | **C2** | **0.016** | **0.028** | **0.010** | **0.019** |
| **E1** | **E2** | 0.370 | **0.043** | 0.701 | 0.484 |
| **E1** | **C1** | 0.304 | 0.231 | **0.023** | **0.013** |
| **E1** | **C2** | 0.289 | 0.064 | **0.016** | **0.015** |
| **E2** | **C1** | **0.008** | **0.010** | **0.014** | **0.011** |
| **E2** | **C2** | **0.037** | **0.034** | **0.017** | **0.024** |
| **C1** | **C2** | 0.643 | 0.355 | **0.48** | 0.857 |

**Additional table 3: Results and asymptotic significances calculated for CD20 and CD45R quantification in spleen via Mann-Whitney-U test.** P-values < 0.05 were considered to be significant.

| **CD20** | | | | | | | | | | |
| --- | --- | --- | --- | --- | --- | --- | --- | --- | --- | --- |
| **Labels** | | **C1** | | **E1** | | | **C2** | | **E2** | |
| **Min** | | 1085 | | 191 | | | 589 | | 242 | |
| **Q_1_** | | 1102 | | 256 | | | 645 | | 284 | |
| **Median** | | 1293 | | 256 | | | 819 | | 284 | |
| **Q_3_** | | 1331 | | 307 | | | 1142 | | 341 | |
| **Max** | | 1462 | | 322 | | | 1303 | | 358 | |
| **IQR** | | 228 | | 51 | | | 497 | | 57 | |
| **Upper Outliers** | | 0 | | 0 | | | 0 | | 0 | |
| **Lower Outliers** | | 0 | | 0 | | | 0 | | 0 | |
| **CD45R** | | | | | | | | | | |
| **Labels** | | **C1** | | **E1** | | | **C2** | | **E2** | |
| **Min** | | 4606 | | 2193 | | | 4979 | | 2047 | |
| **Q_1_** | | 6382 | | 2201 | | | 6255 | | 3137 | |
| **Median** | | 6580 | | 2320 | | | 6366 | | 3257 | |
| **Q_3_** | | 7677 | | 2497 | | | 6507 | | 3464 | |
| **Max** | | 8031 | | 3191 | | | 6518 | | 4192 | |
| **IQR** | | 1294 | | 297 | | | 252 | | 328 | |
| **Upper Outliers** | | 0 | | 1 | | | 0 | | 1 | |
| **Lower Outliers** | | 0 | | 0 | | | 1 | | 1 | |
| **P-values** | | | | | | | | | | |
|  | **E1 VS E2** | | **E1 VS C1** | | **E1 VS C2** | **E2 VS C1** | | **E2 VS C2** | | **C1 VS C2** |
| CD20 | 0.344 | | 0.009 | | 0.009 | 0.009 | | 0.009 | | 0.117 |
| CD45R | 0.175 | | 0.009 | | 0.009 | 0.009 | | 0.009 | | 0.251 |

**Additional figure 1 Supplementary immunohistochemical stainings.** Part of a lymph follicle of C0 showing in **(a)** the B-cell marker CD45R and in **(b)** CD20. Representative immunohistochemical staining of Caspase-3 is shown in **(c) – (f)**. Positive, apoptotic cells appear in brown, there is no counterstaining visible. In comparison to the controls C1 **(c)** and C2 **(e)** there are hardly any apoptotic cells detectable in the therapy groups E1 **(d)** and E2 **(f)**. Immunohistochemical double staining of GFAP (violet) and Caspase-3 (brown) is shown in **(g)** and **(h).** Most of the apoptotic cells are astrocytes. There are much more apoptotic astrocytes detectable in controls **(g)** in comparison to the therapy group **(h).** Infiltrates with T- and B-cells are very sparse in this animal model. CD3 positive T-cells and CD19 positive B-cells were only detected in minor traces in the meninges. Representative pictures of T-cells in E1 are given in **(i)** and of B-cells in **(j)**. Red arrows point at the very few positive stained cells in dark brown. Scale bars represent 100 μm.

**Additional figure 2 Quantification of additional myelin markers MBP and MOG and quantification of CD20 and CD45R positive cells in spleen.** All myelin quantifications of additional markers show comparable results to PLP quantification. For MBP there are significant differences between E2 and C1 (ipsi.: p < 0.008; con.: p < 0.010) and E2 and C2 (ipsi.: p < 0.037; con.: p < 0.034) on both sides **(a)** and **(b)**. There are significant differences between all experimental and control groups on both sides for MOG **(c)** and **(d)** with p values ranging from 0.011 to 0.024. For exact p-values for all groups see additional table 2. For both B-cell markers, CD20 **(e)** and CD45R **(f)**, there is a significant difference detectable between all experimental groups and control groups (p < 0.009, see also additional table 3). There is no significant difference between E1 and E2 or C1 and C2. For these results a representative set of n=5 lymph follicle per group was quantified.
