# Supplementary figures and images for: Anti-CD20 treatment effectively attenuates cortical pathology in a rat model of widespread cortical demyelination
Source: J Neuroinflammation. 2021 Jun 15;18:138. doi: 10.1186/s12974-021-02189-w (PMC8207776; doi:10.1186/s12974-021-02189-w)

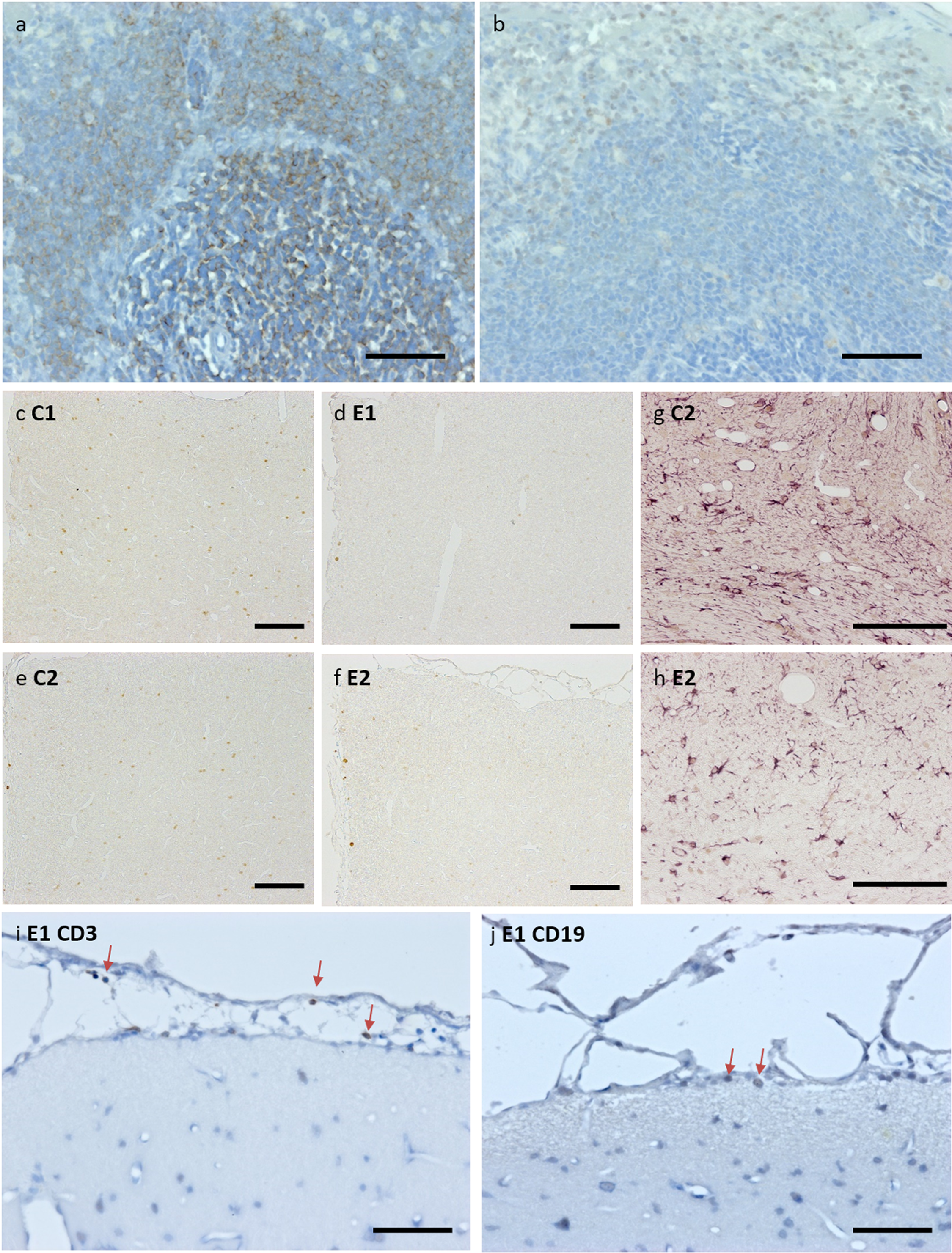

Supplement: Supplementary file 2 — Additional file 2: Additional Figure 1. Supplementary immunohistochemical stainings. Part of a lymph follicle of C0 showing in (a) the B-cell marker CD45R and in (b) CD20. Representative immunohistochemical staining of Caspase-3 is shown in (c) – (f). Positive, apoptotic cells appear in brown, there is no counterstaining visible. In comparison to the controls C1 (c) and C2 (e) there are hardly any apoptotic cells detectable in the therapy groups E1 (d) and E2 (f). Immunohistochemical double staining of GFAP (violet) and Caspase-3 (brown) is shown in (g) and (h). Most of the apoptotic cells are astrocytes. There are much more apoptotic astrocytes detectable in controls (g) in comparison to the therapy group (h). Infiltrates with T- and B-cells are very sparse in this animal model. CD3 positive T-cells and CD19 positive B-cells were only detected in minor traces in the meninges. Representative pictures of T-cells in E1 are given in (i) and of B-cells in (j). Red arrows point at the very few positive stained cells in dark brown. Scale bars represent 100 μm. [file 12974_2021_2189_MOESM2_ESM.tif]

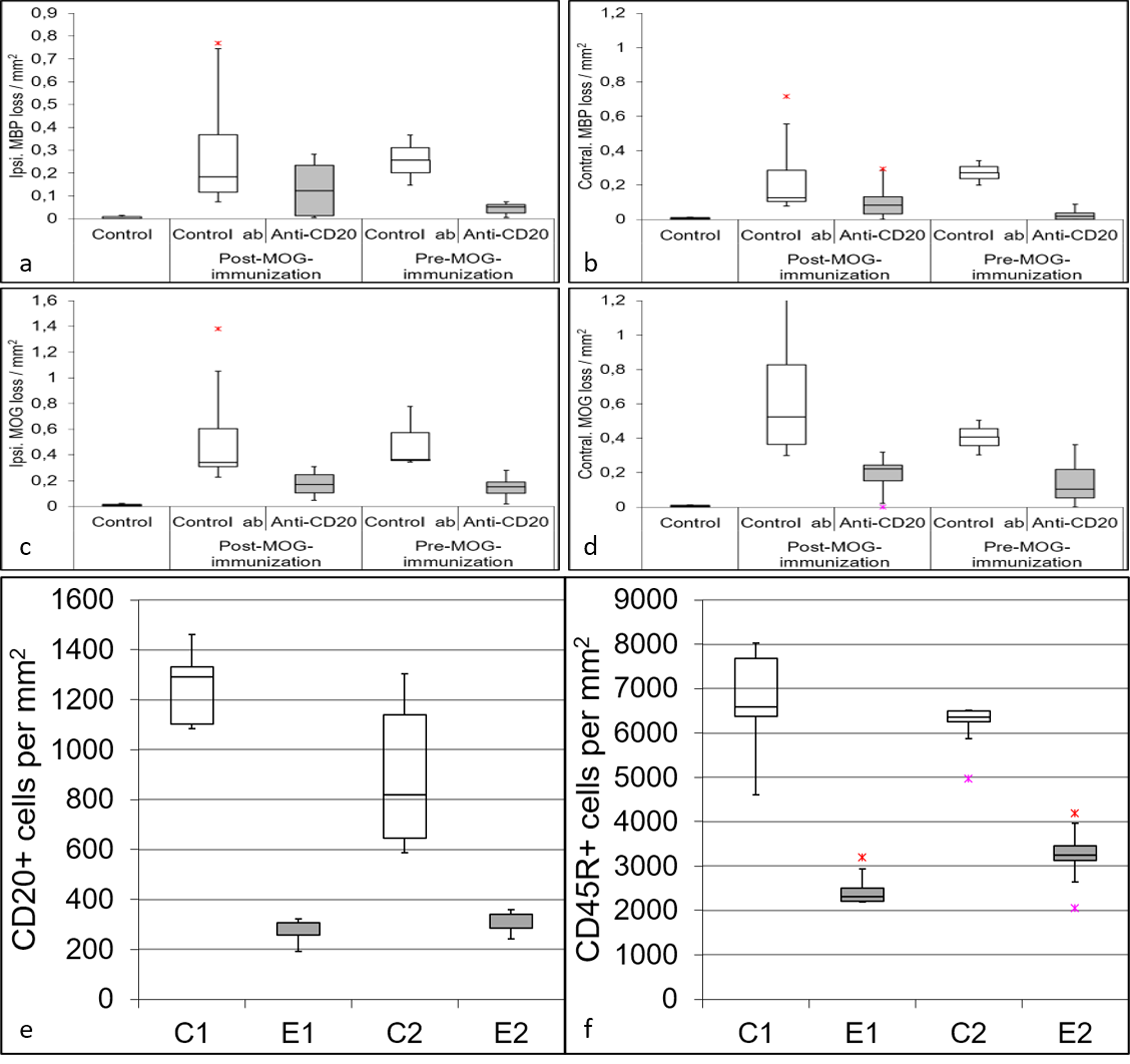

Supplement: Supplementary file 3 — Additional file 3: Additional Figure 2. Quantification of additional myelin markers MBP and MOG and quantification of CD20 and CD45R positive cells in spleen. All myelin quantifications of additional markers show comparable results to PLP quantification. For MBP there are significant differences between E2 and C1 (ipsi.: p < 0.008; con.: p < 0.010) and E2 and C2 (ipsi.: p < 0.037; con.: p < 0.034) on both sides (a) and (b). There are significant differences between all experimental and control groups on both sides for MOG (c) and (d) with p values ranging from 0.011 to 0.024. For exact p-values for all groups see additional Table 2. For both B-cell markers, CD20 (e) and CD45R (f), there is a significant difference detectable between all experimental groups and control groups (p < 0.009, see also additional Table 3). There is no significant difference between E1 and E2 or C1 and C2. For these results a representative set of n=5 lymph follicle per group was quantified. [file 12974_2021_2189_MOESM3_ESM.tif]
